# Simultaneous Salmonella septic arthritis and naïve tricuspid valve endocarditis: A case report

**DOI:** 10.22088/cjim.15.3.542

**Published:** 2024-08-01

**Authors:** Mahnaz Arian, Farideh Najm Sarvari, Moein Mohebbi, Marzieh Kazerani

**Affiliations:** 1 Department of Infectious Diseases, Faculty of Medicine, Mashhad University of Medical Sciences, Mashhad, Iran; 2 Faculty of Medicine, Mashhad University of Medical Sciences, Mashhad, Iran

**Keywords:** Salmonella, Septic arthritis, Infective endocarditis, Tricuspid valve

## Abstract

**Background:**

Salmonella osteoarticular involvement is a rare complication, occurring in about 2% of the cases. Septic arthritis is exceedingly rare, involving only 0.2 % of all salmonellosis patients. Endocarditis is another complication that occurs in less than 0.8 % of cases. These complications are more likely to happen among immunocompromised patients.

**Case Presentation:**

We report a previously healthy 25-year-old man who presented with left limb pain. He had been treated for brucellosis ten days earlier by his primary care physician. Arthrocentesis and subsequent hip-joint biopsy confirmed septic arthritis due to Salmonella. However, he was unresponsive to the treatment. We found no underlying immunosuppression. A trans-esophageal echo was performed due to the continued fever and positive blood cultures. It revealed Salmonella endocarditis of the naïve tricuspid valve. He was treated via arthrotomy and antimicrobials for four weeks. Follow-up after 20 months showed no underlying immunosuppression.

**Conclusion:**

This case highlights that in patients with positive Salmonella blood cultures and a focus of infection compatible with Salmonellosis but unresponsive to treatment, searching for other foci of infection is necessary. Furthermore, physicians in endemic areas of brucellosis should consider other differential diagnoses in patients with fever and limping because any delay in diagnosing Salmonella septic arthritis can destroy the joint space with lifelong discomfort.

Salmonella is a facultative gram-negative rod that belongs to the Enterobacteriaceae family. In the US, it causes 1.2 million illnesses and 450 deaths each year (1). It usually presents with gastrointestinal complaints, but rarely, it can cause extraintestinal manifestations. Osteoarticular involvement is unusual, occurring in about 2% of the cases. Septic arthritis is even rarer, involving only 0.2 % of all Salmonellosis (2). Endocarditis is another complication that occurs in less than 0.8 % of cases (3, 4). Extraintestinal complications are more likely to happen among immunocompromised patients. We describe a case with simultaneous right-sided naïve-valve endocarditis and Salmonella septic arthritis (SSA), which was initially mistaken for brucellosis. Unfortunately, this delay in treatment caused joint destruction.

## Case Presentation

A 25-year-old, previously healthy man was brought to the emergency department with an inability to stand on his right foot. He also complained of pain, swelling, and vague heaviness in his right groin. He mentioned that the pain started three weeks earlier after a swimming episode and got aggravated until it caused claudication and a subsequent bed-ridden state. 

He reported an appetite loss, 5-Kgs weight loss, fever (only detected on the first day of pain onset), and no night sweats. Past medical and travel history was unremarkable. Ten days before this admission, his primary care physician prescribed rifampin and doxycycline with a probable diagnosis of brucellosis. He was also using NSAIDs for pain management. He was a construction worker, non-smoker, and non-drinker but was addicted to Naswar (smokeless tobacco). On examination, he was lying in a bed with his right hip flexed, reluctant to be examined. His vital signs were stable (blood Pressure: 120/80 mm/Hg, pulse rate: 80 bpm, respiratory rate: 16 cycles per minute, temperature: 37.1 degrees of Celsius rectally). General examination findings, including heart sounds, were unremarkable. The right hip was slightly red and swollen, with severe tenderness on palpation and a restricted range of motion. Lymphadenopathies on femoral and inguinal sites were detected. He was diagnosed with septic arthritis, admitted by the emergency medicine department, and started on Cefazolin. White blood count: 8500/mm^3^ (PMN: 69%, LMN: 20%, Mix: 9.9%), hemoglobin: 13.9 mg/dL, hematocrit: 40%, platelets: 577/mm^3^, CRP: 54 mg/L, ESR: 108 mm/hr.

**Figure 1:**
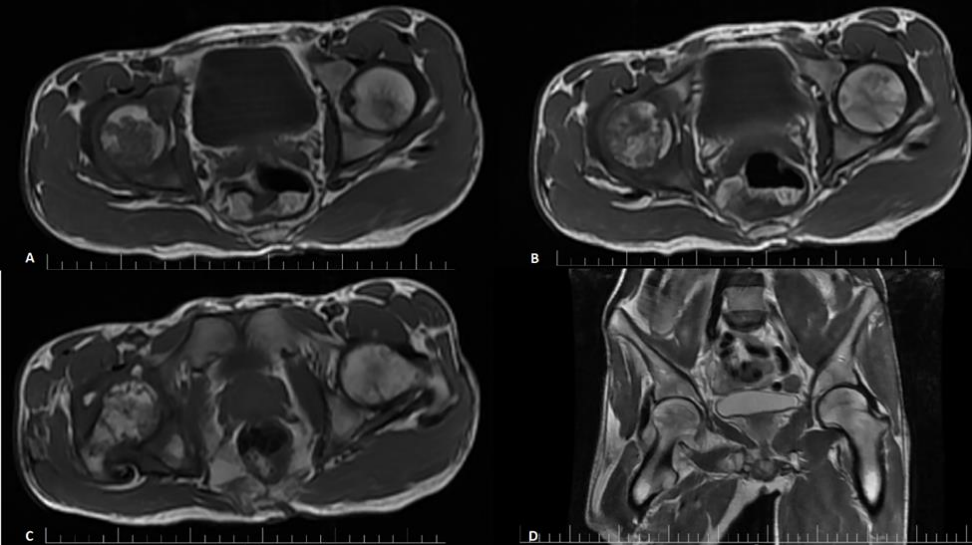
A, B, C: transverse section in an ascending fashion from the femur towards the base of the pelvis (T1W). D: The coronal section of the hip joints shows destruction and irregularity.

Right hip ultrasound reported increased synovial thickening with mild synovial effusion and some reactive lymph nodes in the inguinal region. MRI findings showed “Right hip irregular surface, abnormal fluid signal with filling defect suggesting loose body, and mild periarticular soft tissue edema and swelling” (figure 1). The arthrocentesis specimen was found positive for Salmonella. Antibiogram showed sensitivity to Cefotaxime, Cefepime, Ciprofloxacin, Ceftriaxone, and Ceftazidime. The treatment changed to Ceftriaxone. Arthrotomy was planned, and synovial biopsy was again positive for Salmonella, serogroup D, with the same antibiotic sensitivity pattern. Pathology reports of a synovial specimen of the right hip showed acute inflammation in the background of chronic synovitis.

After no significant response to the treatment, infectious diseases consult was requested. Based on the consult, the treatment changed to Cefepime (1 gram TDS). Two separate blood cultures, stool exam and culture, urinalysis and cultures, hemoglobin electrophoresis, HIV status, and EKG were requested to rule out predisposing conditions. The blood culture was positive for Salmonella serogroup D and the antibiogram showed Cefepime, Cefotaxime, Ceftazidime, Ceftriaxone, and Ciprofloxacin sensitivity but the rest of the results of the mentioned tests were unremarkable. We asked for a rheumatology consult, and the results of the ordered tests were as follows: negative for HLA-B27, rheumatoid factor: 3.4 (up to 30 is normal), Anti-CCP: 2.4 (<12 is negative), ANA: 3.4 (<12 is negative), Anti-DS DNA: 37.2 (>24 is positive), and negative Wright and 2ME. Transthoracic echo (TTE) only showed mild mitral regurgitation and mild tricuspid regurgitation. Still, due to a high index of suspicion, a trans-esophageal echo (TEE) was performed, which reported a filamentous hypo-echo structure (length: 9-10mm) attached to the atrial side of the anterior leaflet of the tricuspid valve. The Cefepime regimen continued, and after seven days of treatment, ESR declined to 95 mm/hr, and follow-up TEE reported a previous structure with reduced length (4.5mm). After four weeks of antimicrobial therapy, the patient was discharged with an ESR of 69 mm/hr and no pain. After 20 months of follow-up, he complains of mild right hip discomfort but is otherwise healthy. 

## Discussion

Genus Salmonella (belongs to the Enterobacteriaceae family) can cause diverse clinical symptoms considering the subtype of the organism and the immune state of the host (5). Based on the clinical syndrome, S.enterica subgroup I is divided into typhoidal (cause enteric fever) and non-typhoidal salmonella (NTS), which cause self-limiting enteritis in an immunocompetent host. In immunocompromised patients, NTS usually invades beyond the gastrointestinal tract and drives extraintestinal involvement (5). As a result, any focal infection (i.e., endocarditis, soft tissue infection, UTIs, etc.) due to NTS should raise suspicions of an underlying condition that affects normal immune homeostasis (2). Extraintestinal involvement occurs in about 2% of all human Salmonella infections. Osteoarticular complications are exceedingly rare (0.8%), including reactive arthritis (the most frequent), osteomyelitis, and septic arthritis (2, 6). Septic arthritis causes 0.2% of osteoarticular complications and is frequently caused by *S.Choleraesuis* (more frequent) and *S.typhimurium* (better prognosis) (2, 7). Osteomyelitis and septic arthritis are usually monoarticular, while reactive arthritis involves more than one joint. Morgan et al. claimed that the usual site for septic arthritis is the knee, followed by the hip and shoulder (7). The mean age of Salmonella septic arthritis among immunocompromised patients is 37-42 years in two different studies (8, 9). Several risk factors are identified for Salmonella septic arthritis such as age ( less than 5, more than 65 years old), trauma, surgery (prosthetic joint), systemic lupus erythematous (SLE), corticosteroid use, human immunodeficiency virus (HIV), sickle cell anemia (SCA), neoplasm, chronic renal disease, chronic liver failure, chronic alcoholism, rheumatoid arthritis, chronic obstructive pulmonary disease, avascular necrosis (AVN) (6, 10-12). 20% of all Salmonella bacteremia occurs in SLE patients; it even might be the first manifestation of the disease (8). *Panels et al*. present ten SLE cases with NTS infection, all of which were complicated with extraintestinal involvement (11). Diagnosis of this complication in patients with previous SLE is difficult and might be delayed due to the clinical similarity of SLE flare with infection (8). CRP levels might help differentiate these two conditions (mean CRP of 42 in infection in contrast to 8.8 in a flare) (8). AVN in SLE patients is probably the most predisposing factor for SSA. SLE-related SSA mostly affects the hip (followed by the knee), and Salmonella serotype B is more frequent than S.Choleraesuis (10). SSA in African individuals should raise suspicions of HIV (5). Corticosteroid use can cause AVN, and both can predispose patients to SSA. The essential role of complement in clearing Salmonella might explain why liver failure, chronic alcoholism, and renal disease predispose patients to complicated Salmonella infection (5). Imaging is not necessary to diagnose septic arthritis. Still, it yields more accurate joint aspiration, local information detection, the degree of joint destruction, and ruling out simultaneous pathology (like accompanying osteomyelitis, AVN, etc.). Plain radiographs and ultrasonography are usually chosen as initial assessment modalities, while MRI and scintigraphy provide more details of ongoing information. About 1/3 of patients with SSA have simultaneous osteomyelitis, especially those with SCA, highlighting the role of imaging in the management of these patients (7). For treatment, 4-6 weeks of intravenous antibiotic therapy with or without arthrocentesis is recommended. Arthrotomy is considered if primary treatment fails. Trimethoprim-sulfamethoxazole, third-generation cephalosporins, and fluoroquinolones are suggested (10).

1-6% of all Salmonella infections can be complicated by cardiac involvement (4); This can occur as myocarditis, pericarditis, prosthetic-valve endocarditis, and mycotic aneurysm (3). Salmonella infective endocarditis (SIE) accounts for 0.01-0.8% of extraintestinal complications and mostly affects the mitral valve, followed by the aortic valve (3). 25% of SIEs are mural endocarditis with a higher mortality rate (3). Almost 75% of cases suffer predisposing cardiac abnormalities, and like SSA, immunosuppression is a risk factor (13). Presentation is similar to other usual causes of endocarditis (fever, chills, hepatosplenomegaly, and petechia), and only about 30% recall a herald gastrointestinal symptom (3). Usual serotypes are* S*.*Choleraesuis*, *S.typhimurium*, and *S.enterica (13)*. SIE could be complicated by valve or cusp rupture, valve ring abscess, atrioventricular wall rupture, and emboli formation (14). Over time, mortality decreased from 28% to 13.3%, and it is almost the same between naïve-valve versus prosthetic-valve SIE (3). Appropriate antimicrobial regimen and treatment duration have not been well established in the literature, but extended treatment duration is generally recommended. Surgery should be considered if medical treatment fails. 

Negative culture for Salmonella should be repeated in a patient with immunosuppression and fever of unknown origin. Detection of Salmonella bacteremia is a convincing clue, but negative cultures are not conclusive since blood culture sensitivity is only about 40-50% (4, 5). Bone marrow culture has a higher sensitivity of 80-90% (4). 

Simultaneous SSA and SIE were previously reported in a 65-year-old immunocompromised (end-stage lung squamous cell carcinoma) male with aortic valve involvement and septic arthritis of both hips (15). In such cases, the heart is the primary source of septic arthritis. However, to the best of our knowledge, isolated right-sided heart involvement and simultaneous septic arthritis have not previously been reported.

This case highlights that searching for other foci of infection is necessary for patients with positive Salmonella blood cultures and a focus of infection compatible with Salmonellosis but unresponsive to treatment. Furthermore, physicians in endemic areas of brucellosis should consider other differential diagnoses in patients with fever and limping because any delay in diagnosing Salmonella septic arthritis can destroy the joint space with lifelong discomfort.

## References

[1] Centers-for-Disease-Control-and-Prevention CDC-Salmonella-Factsheet 2016.

[2] Ajmera A, Shabbir N (2022). StatPearls.

[3] Cheng WL, Li CW, Li MC (2016). Salmonella infective endocarditis. J Microbiol Immunol Infect.

[4] Robson C, O'Sullivan MVN, Sivagnanam S (2018). Salmonella enterica Serovar Typhi: An Unusual Cause of Infective Endocarditis. Trop Med Infect Dis.

[5] Sanderson KE, Liu SL, Tang L, Johnston RN (2015). Salmonella Typhi and Salmonella Paratyphi A. InMolecular medical microbiology.

[6] Tassinari AM, Romaneli MTDN, Pereira RM, Tresoldi AT (2019). Septic arthritis caused by Salmonella enterica serotype Rubislaw: A case report. Rev Soc Bras Med Trop.

[7] Morgan MG, Forbes KJ, Gillespie SG (1990). Salmonella septic arthritis: a case report and review. J Infect.

[8] Kedzierska J, Piatkowska-Jakubas B, Kedzierska A (2008). Clinical presentation of extraintestinal infections caused by non-typhoid Salmonella serotypes among patients at the University Hospital in Cracow during an 7-year period. Pol J Microbiol.

[9] Chen JY, Luo SF, Wu YJ, Wang CM, Ho HH (1998). Salmonella septic arthritis in systemic lupus erythematosus and other systemic diseases. Clin Rheumatol.

[10] Pablos JL, Aragon A, Gomez-Reino JJ (1994). Salmonellosis and systemic lupus erythematosus. Report of ten cases. Br J Rheumatol.

[11] Lo IF, Chang HC (2018). Salmonella septic arthritis in a patient with a hip implant: a case report. Int J Gerontol.

[12] Khan JA, Ali B, Masood T (2011). Salmonella typhi infection: a rare cause of endocarditis. J Coll Physicians Surg Pak.

[13] Laganà P, Delia S, Dattilo G, Mondello C, Ventura Spagnolo E (2017). A case of infective endocarditis due to Salmonella enterica phagetype 35. First report. Clin Ter.

[14] García M, García N, Striebeck P, Cejas D, Rodríguez V (2016). Endocarditis and arthritis caused by extended spectrum β-lactamase-producing non-Typhi Salmonella. Rev Chilena Infectol.

